# Induction of psoriasis- and atopic dermatitis-like phenotypes in 3D skin equivalents with a fibroblast-derived matrix

**DOI:** 10.1038/s41598-023-28822-7

**Published:** 2023-01-31

**Authors:** Bianka Morgner, Jörg Tittelbach, Cornelia Wiegand

**Affiliations:** Department of Dermatology, University Hospital Jena, Friedrich Schiller University Jena, Erfurter Straße 35, 07743 Jena, Germany

**Keywords:** Biological techniques, Cell biology, Diseases, Molecular medicine

## Abstract

Skin homeostasis is a complex regulated process relying on the crosstalk of keratinocytes, fibroblasts and immune cells. Imbalances of T-cell subsets and the cytokine environment can lead to inflammatory skin diseases such as psoriasis (Ps) and atopic dermatitis (AD). Modern tissue engineering provides several in vitro models mimicking Ps and AD phenotypes. However, these models are either limited in their pathological features, life span, sample availability, reproducibility, controlled handling or simplicity. Some models further lack intensive characterization as they solely focus on differentiation and proliferation aspects. This study introduces a self-assembly model in which the pathological T-cell-signalling of Ps and AD was simulated by subcutaneous Th1 and Th2 cytokine stimulation. The self-established dermal fibroblast-derived matrices of these models were hypothesized to be beneficial for proximal cytokine signalling on epidermal keratinocytes. Comprehensive histological and mRNA analyses of the diseased skin models showed a weakened barrier, distinct differentiation defects, reduced cellular adhesion, inflammation and parakeratosis formation. A keratin shift of declining physiological cytokeratin-10 (CK10) towards increasing inflammatory CK16 was observed upon Th1 or Th2 stimulation. Antimicrobial peptides (AMPs) were upregulated in Ps and downregulated in AD models. The AD biomarker genes *CA2*, *NELL2* and *CCL26* were further induced in AD. While Ps samples featured basal hyperproliferation, cells in AD models displayed apoptotic signs. In accordance, these well-controllable three-dimensional in vitro models exhibited Ps and AD-like phenotypes with a high potential for disease research and therapeutic drug testing.

## Introduction

Psoriasis (Ps) and atopic dermatitis (AD) are chronic inflammatory skin diseases, occurring worldwide with high prevalence^1,2^. Patients do not only suffer from scaling, itching, redness, inflammation and infections (AD), but also have a high mental burden and impaired quality of life. Beside skin symptoms and depression, patients with psoriasis have a higher rate of comorbidities like metabolic syndrome, arteriosclerosis, myocardial infarction, cerebral insult. In atopic dermatitis, common comorbidities include allergic asthma, allergic rhinoconjunctivitis, food allergies, infections (bacterial, viral) and even coronary artery disease^3,4^. Ps and AD share some similarities in their macroscopic phenotypes and both are classified as T helper (Th) cell-derived disorders. The subsets of these lymphocytes responsible for the pathological onsets however differ. While Ps is triggered by excessive amounts of Th1 and Th17 cells, AD develops upon a predominance of Th2 subsets. Th1 and Th17 cells release cytokines such as IL-17A, IL-22 and TNF-α as a major source of Ps disease development^5^. These T-cell-derived cytokines stimulate the release of inflammatory mediators by epidermal keratinocytes and result in abnormal differentiation and hyperproliferation. Recent studies suggest IL-22 to contribute to Ps onset and progression. Elevated mRNA transcripts of the interleukin have been found in patient’s skin biopsies of psoriatic lesions^6^. IL-22 affects keratinocytes by proliferation induction but inhibition of differentiation^7^. IL-6, IL-23 and IL-1 are found to be increased in psoriatic skin and are known stimuli for Th17 polarization, which further sustains the inflammatory cycle^8,9^. In AD, Th2 cells secrete IL-4, IL-13 and IL-31 acting as main drivers of barrier defects by reduction of differentiation markers such as filaggrin (FLG), loricrin (LOR) and involucrin (IVL)^10,11^. On molecular levels, the diseases can be distinguished via typical signatures of antimicrobial peptides (AMPs). AMPs such as psoriasin (S100A7), human beta-defensin 2 (encoded by the *DEFB4* gene), cathelicidin LL37, PI3 (elafin) and LCN2 (lipocalin-2) are strongly induced in Ps but downregulated in acute AD^12–16^. Due to low AMP levels in atopic skin, patients are at high risk of bacterial infections^17^. In contrast, excessive AMP secretion in psoriatic skin provides a defence mechanism protecting the impaired cutaneous barrier against microbial infections^18^. In addition, AD biomarker genes have been identified such as carbonic anhydrase (CAII/*CA2*), neural epidermal growth factor-like like 2 (NELL2) and the C–C motif chemokine ligand 26 (CCL26)/eotaxin-3^19,20^. The latter is chemoattractant to eosinophilic granulocytes leading to strong infiltrates of these cells in AD skin^20^. Besides, eosinophils participate in allergic reactions explaining the high susceptibility of AD patients towards allergies^21^. Despite much is known about molecular mechanisms behind Ps and AD, it is not clear what initially triggers the imbalances of Th cells. Disease heterogeneities seen in Ps and AD patients as well as involvement of multiple signalling pathways, proteins and stimuli impedes with treatment of the disorders. Still, both, Ps and AD, are not permanently curable with the therapies and medications currently available. Hence, it is of importance to further study the pathological processes involved in disease onset and progression. By understanding the detailed pathomechanism, therapeutic approaches can be adapted for a target-specific treatment. However, this requires the availability of well-characterized test systems simulating in vivo-like conditions as much as possible while being controllable and simple in use. Modern techniques of tissue engineering provide promising tools for in vitro studies of cutaneous structures, functions and cellular interactions. In addition, pathological phenotypes can be introduced in these 3D systems, thereby allowing the analysis of cutaneous diseases in a way that closely resembles in vivo-like conditions. Various skin equivalents have been established in order to mimic psoriasis and atopic dermatitis. The most simple ones rely on keratinocytes forming a reconstructed human epidermis (RHE)^22–27^. Despite resembling several disease-features, the communication between keratinocytes and fibroblasts and the involvement of these mesenchymal cells is neglected in the RHE models. While epidermal cells are predominantly affected in Ps and AD, it has been shown that fibroblasts are involved as well^28–31^. So far, de-epidermized dermis (DED) models use dermis biopsies from donors with the addition of primary keratinocytes for epidermal layer generation. In this way, Ps models have been established showing a typical increase in AMPs and inflammatory features^32^. However, their use is limited by the availability of DED samples, which further are hampered by high donor variation. In addition, the generation of these skin models is rather time-consuming. Another group directly used either psoriatic keratinocytes or fibroblasts in a self-assembly model, which resulted in hyperproliferation and abnormal differentiation. Other disease-related aspects were not investigated here^33^. This model also depends on the availability of diseased donor cells, which are probably more rarely obtained than healthy donor cells. Therefore, many researchers often use recombinant cytokines to simulate the pathological function of T-cells. Another model of interest is a collagen-based model. Here, fibroblasts are seeded in an artificial dermal matrix using rat collagen underneath the epidermis^34^. Using collagen from animal sources might lack human proteins that are part of the extracellular matrix (ECM). In addition, under in vivo conditions, T-cells reside within the dermis and may also infiltrate the epidermal layers bringing them in close proximity to epidermal cells^35^. Skin equivalents on collagen basis often display a thick dermal compartment. The disease-induction happens upon cytokine supplementation in the medium below. This results in a huge gap between the cytokines in the medium and the epidermal compartment on top of the collagen gels, thus, impeding the proximal regulation. Another drawback is the high contractility of animal collagen depending on the used concentration, which might result in poor epidermis formation^36^. In addition, El Ghalbzouri et al*.* showed that collagen-based models are not suitable for the long-term use, which might be a necessity for treatment experiments of Ps and AD models^36^. In contrast, self-assembly models using fibroblast-derived dermal matrices were shown to have a prolonged lifespan and seem to be superior by creating a dermal compartment enriched with human ECM proteins^36–38^.

In respect to these previous works, this study introduces Ps and AD models based on a fibroblast-derived matrix with an epidermis generated from primary keratinocytes and combining them with recombinant cytokine stimulation for simulation of pathological T-cell signalling. By this means, reproducible, and in vivo-like Ps and AD models have been established that are controllable yet free of some of the afore mentioned limitations. Furthermore, an in-depth analysis was done to validate the potential of these Ps and AD models and illustrate disease-like features.

## Results

### Characteristic histological alterations in disease-associated skin models

**Figure 1:**
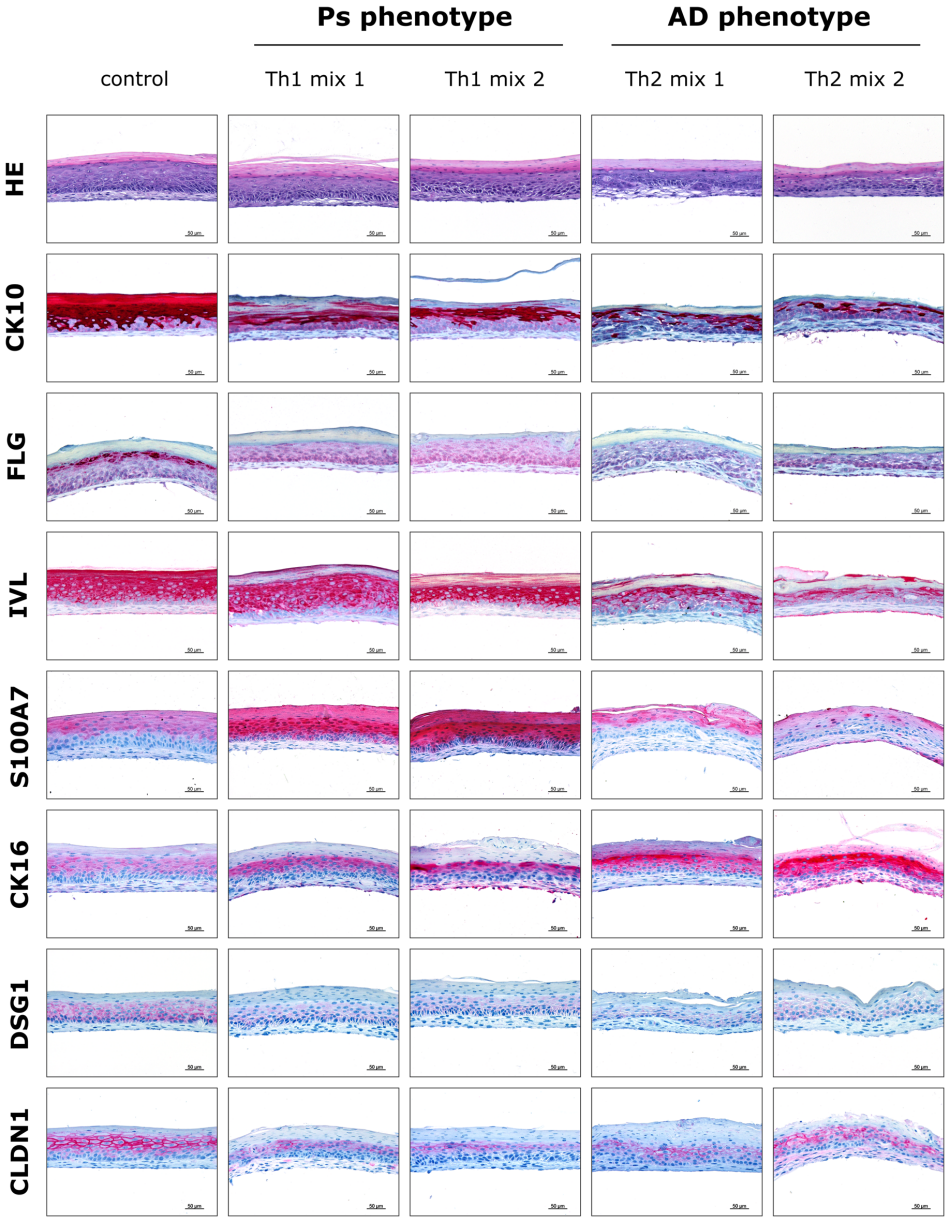
Histological alterations in disease-associated skin models. Microanatomy of the skin models was revealed by HE staining. IHC staining was used for visualization of differentiation markers/structural proteins (CK10, FLG, IVL, CK16), antimicrobial peptides (S100A7) and cellular contact proteins (DSG1, CLDN1) of skin equivalents stimulated with Th1 or Th2 cytokine mixes compared to an unstimulated physiological control. Scale bar: 50 µm; pictures are representatives of n = 2 experiments with a total of 4 skin models (keratinocytes derived from one donor).

In vitro mimicking of disease-associated phenotypes in 3D skin models was introduced upon subcutaneous stimulation with cytokines related to Th1/Th17 (briefly named as Th1 mix) and Th2 signalling. As initial validation of disease induction, the skin models were histologically analysed by immunohistochemistry (IHC) of specific differentiation markers and other proteins relevant for evaluation of disease phenotypes. Haematoxylin & eosin (HE) staining was performed in order to evaluate the morphological quality of the skin models. HE-stained skin models showed a well-layered microanatomy of a fine dermal compartment and a differentiated epidermis terminating in a *stratum corneum*. IHC staining revealed distinct alterations characteristic for the respective cutaneous disorder (Fig. 1). Stimulation with Th1 cytokine mixes (IL-17A, IL-6, IL-22, IL-1α ± TNF-α) for Ps induction and Th2 mixes (IL-4, IL-13 ± IL-31) for AD induction resulted in loss of filaggrin (FLG) and reduction of cytokeratin-10 (CK10) levels while involucrin (IVL) was decreased in AD models but less affected in Ps substitutes. Protein expression of psoriasin (S100A7) was enhanced in Ps models but diminished under AD conditions compared to the physiological control. In contrast to healthy skin models, cytokeratin-16 (CK16) levels were increased in both, Ps and AD equivalents. Cellular adhesions were downregulated in disease-associated skin models as revealed by staining of desmosomal contacts via desmoglein-1 (DSG1) and claudin-1 (CLDN1), a transmembrane component of tight junctions (Fig. 1).

### Impaired barrier function in disease-associated skin models

**Figure 2:**
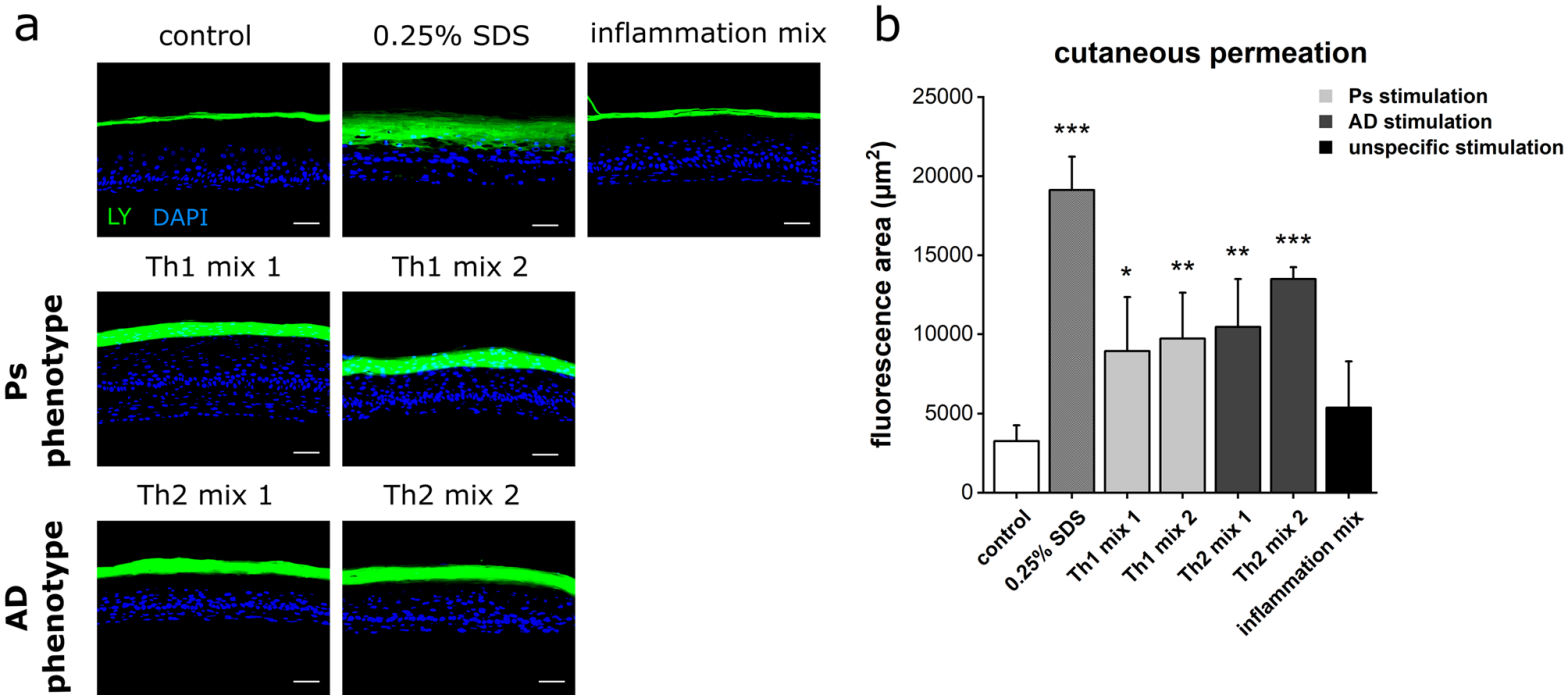
Impaired cutaneous barrier function of disease-associated skin models. (**a**) Penetration of the Lucifer Yellow (LY) fluorescent dye (green) was used to visualize the permeability of skin equivalents stimulated with Th1, Th2 or unspecific inflammatory cytokine mixes compared to an unstimulated physiological negative control. Pretreatment with 0.25% SDS served as permeability control. DAPI (blue) was used for nuclear staining. Scale bar: 50 µm, (**b**) Quantification of LY fluorescence area (mean + SD) of n = 2 experiments with a total of 4 skin models (keratinocytes derived from two different donors). Statistics: one-way ANOVA with Dunett‘s T3 post hoc test; *p* ≤ 0.05 *, *p* ≤ 0.01 **, *p* ≤ 0.001 *** compared to control.

To elucidate the cutaneous integrity and barrier function of Ps and AD models, a Lucifer Yellow assay was implemented. Lucifer Yellow (LY), a fluorescent dye and permeation marker, was topically applied to the skin models and its penetration depth was analysed (Fig. 2). The fluorescent dye penetrated through the *stratum corneum* of physiological controls as well as in Ps and AD skin models. Unlike the positive control exposed to 0.25% SDS where LY was detected in subrabasal regions, the dye accumulated in the cornified layers of all other skin equivalents. However, the horny layers of the diseased models appeared more permeable to LY visible as a wider fluorescent band compared to the thin accumulation line seen in the healthy control (Fig. 2a). To investigate whether a clear correlation can be drawn between barrier defects and specific disease-associated cytokines as combined in Th1 and Th2 cocktails, an additional rather unspecific cocktail came into use. The stimulation with this inflammation mix containing TNF-α and INF-γ did not affect the barrier function since the fluorescent dye accumulated in the same manner as observed in the control (Fig. 2a). Quantification of the LY penetration underlined the microscopical observations. Stimulation with Th1 and Th2 cytokine mixes resulted in significantly increased fluorescence compared to the healthy control. The highest area values were recorded for AD models primed with a combination of IL-4, IL-13 and IL-31 (Th2 mix 2) (Fig. 2b).

### Elevated release of pro-inflammatory mediators by disease-associated skin models

**Figure 3:**
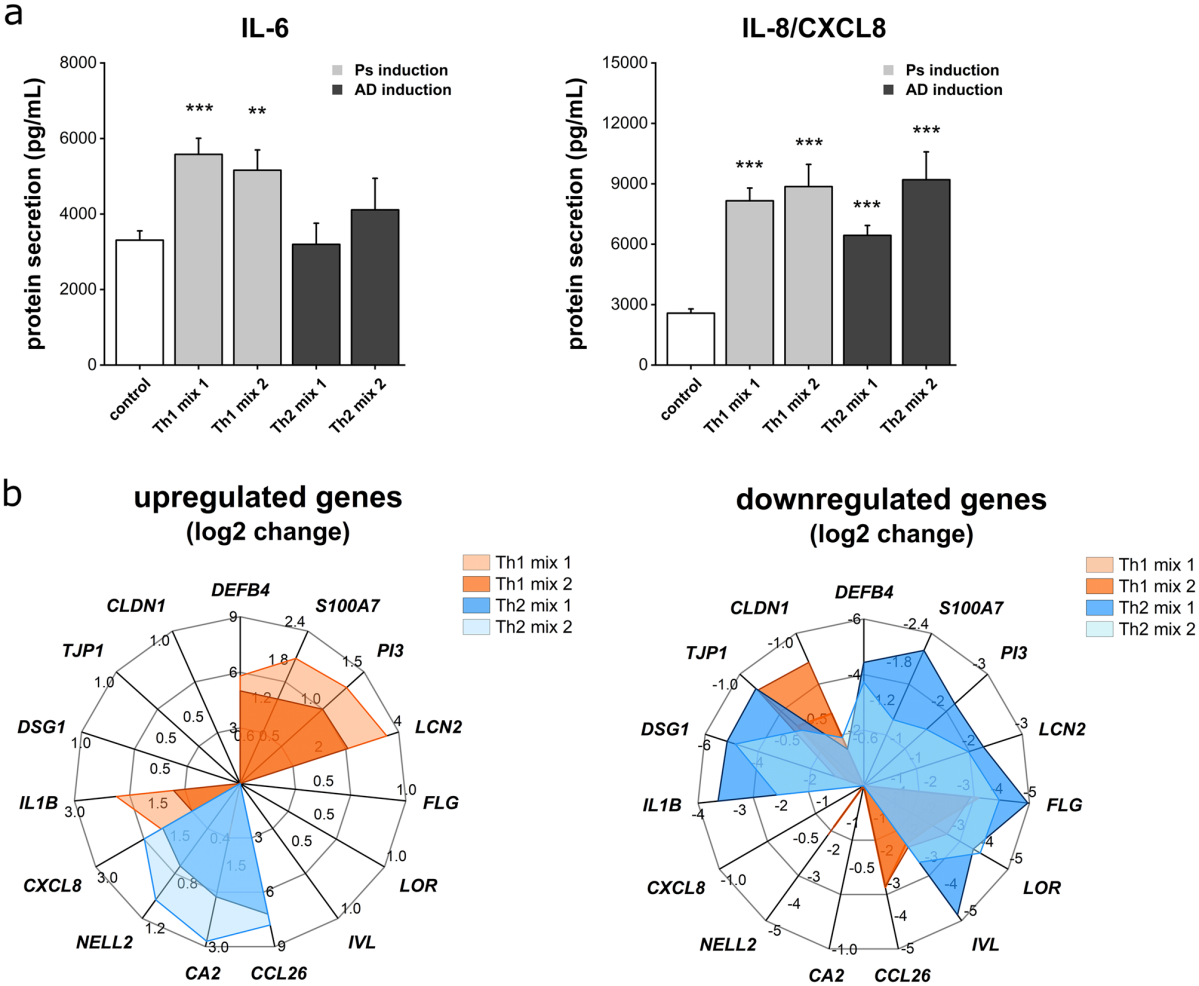
Release of pro-inflammatory mediators and disease-associated gene expression patterns. (**a**) Secretion of the cytokines IL-6 and IL-8/CXCL8 in undernatants of skin equivalents stimulated with Th1 or Th2 cytokine mixes compared to an unstimulated physiological control was measured by ELISA. Values are mean + SEM of n = 2 experiments with a total of 8 skin models (keratinocytes derived from one donor). Statistics: Mann–Whitney U test; *p* ≤ 0.05 *, *p* ≤ 0.01 **, *p* ≤ 0.001 *** compared to control. (**b**) Log2 change of mRNA expression of genes encoding antimicrobial peptides (*DEFB4*, *S100A7*, *PI3*, *LCN2*), structural proteins (*FLG*, *IVL*, *LOR*), chemokines & cytokines (*CXCL8*, *IL1B*, *CCL26*), AD biomarkers (*CA2*, *NELL2*) and protein components of cellular adhesions (*DSG1*, *TJP1*, *CLDN1*) was determined by real-time qPCR. Values are mean of n = 2 experiments with a total of 4 skin models.

In order to investigate the inflammatory features of the Ps and AD substitutes generated in this study, the release of the pro-inflammatory mediators IL-6 and IL-8 was measured by ELISA. Ps stimulation via Th1 mix 1 and 2 significantly increased IL-6 and IL-8 secretion in the undernatants of the psoriatic skin models (Fig. 3a). As for the AD models, IL-6 release was only increased by trend when the stimulation cocktail was supplemented with IL-31 (Th2 mix 2). A potent increase of the IL-8 secretion was observed for the AD equivalents (Fig. 3a).

### Disease-associated gene expression patterns

Expression of mRNA transcripts of various genes was investigated to further characterize the disease skin models. To get an overview which pathways and mRNA levels of certain genes are affected by altering the cytokine environment, genes encoding interleukins, chemokines, differentiation markers, structural proteins important for barrier formation, antimicrobial peptides (AMPs) and cell–cell contact proteins were analysed. It was found that mRNA expression of AMP genes such as *DEFB4* (defensin, beta 4), *S100A7* (psoriasin), *PI3* (elafin) and *LCN2* (lipocalin-2) was significantly increased in Ps-like skin models while potently downregulated in AD-like substitutes. Especially the *DEFB4* gene expression was highly amplified by a 5–6 log2 increase in Ps models and strikingly declined to log2 values of around -4 in AD models compared to the unstimulated healthy control (Fig. 3b, Supplementary Fig. S1). Decreased mRNA expressions of *FLG*, *LOR* and *IVL* were observed in both, Ps- and AD-like skin models (Fig. 3b). As for AD phenotypes, the expression of disease-associated biomarker genes such as *CCL26* (eaotaxin-3), *CA2* and *NELL2* was examined. *CCL26* mRNA levels of AD models exceeded those of healthy controls by a 7–8 log2 change induction. An upregulation was also observed for *CA2* (up to log2 change of 3) and *NELL2* (around a log2 value of 1) transcripts upon Th2 stimulation (Fig. 3b, Supplementary Fig. S1). Regarding inflammatory mediators, the mRNA levels of the chemokine *CXCL8* and the cytokine *IL1B* were monitored. *CXCL8* expression was significantly elevated in both, Ps- and AD-associated skin equivalents. Upon Th1 stimulation, *IL1B* transcription was increased. In contrast, the cytokine gene appeared to be downregulated in AD skin models (Fig. 3b, Supplementary Fig. S1). Another target group aimed at genes encoding proteins involved in the formation of cell–cell adhesions. The mRNA expression of the desmosomal gene *DGS1* was depleted in the both diseased models, with AD models being more negatively affected on transcriptional level than the Ps ones. In addition, the tight junction gene *TJP1* was downregulated under Th1 and Th2 environments as well. On the contrary, *CLDN1* transcripts appeared to be decreased in Ps skin models but were only marginally lower in AD substitutes.

### Hyperproliferation in Ps-like skin models

**Figure 4:**
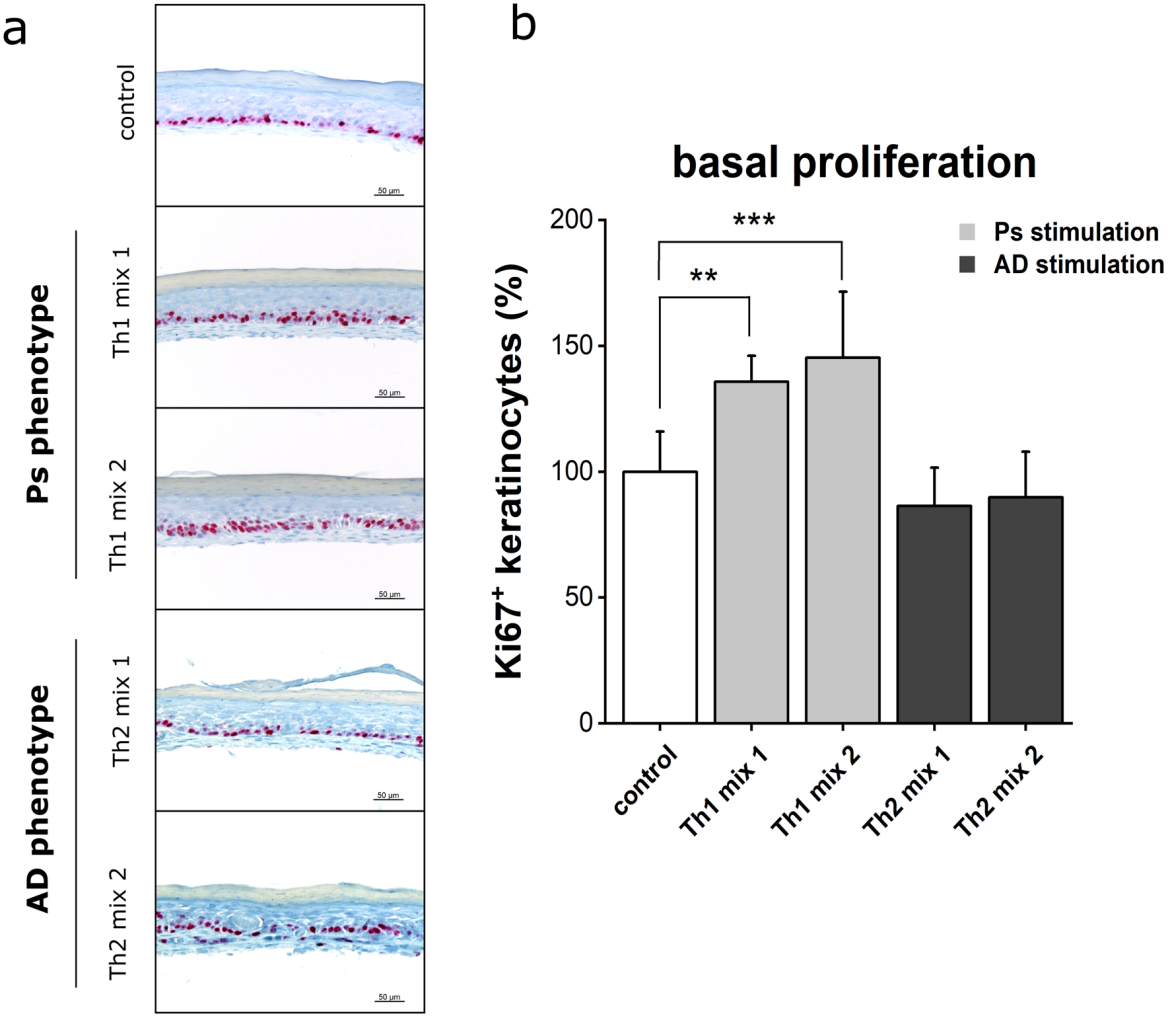
Basal hyperproliferation in psoriasis-like skin models. (**a**) Mitotic cells of skin equivalents (unstimulated physiological control or stimulated with Th1, Th2 cytokine mixes) were visualized using Ki67 immunohistochemistry staining. Scale bar: 50 µm, (**b**) Quantification of Ki67 positive keratinocytes in % to unstimulated control (mean + SD) of n = 2 experiments with a total of 4 skin models (keratinocytes derived from one donor). Statistics: one-way ANOVA with Bonferroni post hoc test; *p* ≤ 0.05 *, *p* ≤ 0.01 **, *p* ≤ 0.001 *** compared to control.

Mitotic events in the *stratum basale* were visualized by IHC staining of the proliferation marker Ki67. Mitosis occurred in each skin equivalent restricted to the basal layer. Ps models were shown to feature a distinctly higher number of Ki67-positive basal cells (Fig. 4a). Cell divisions were quantified by counting the number of Ki67-positive cells per skin equivalent. Stimulation with Th1 cocktails resulted in significantly increased mitotic events in the *stratum basale* of Ps models compared to unstimulated controls or Th2-mediated AD substitutes. Addition of TNF-α to the cytokine mix (Th1 mix 2) further facilitated the mitogenic potential of the stimulation cocktail used for Ps induction (Fig. 4b).

### Parakeratosis in disease-associated skin models

**Figure 5:**
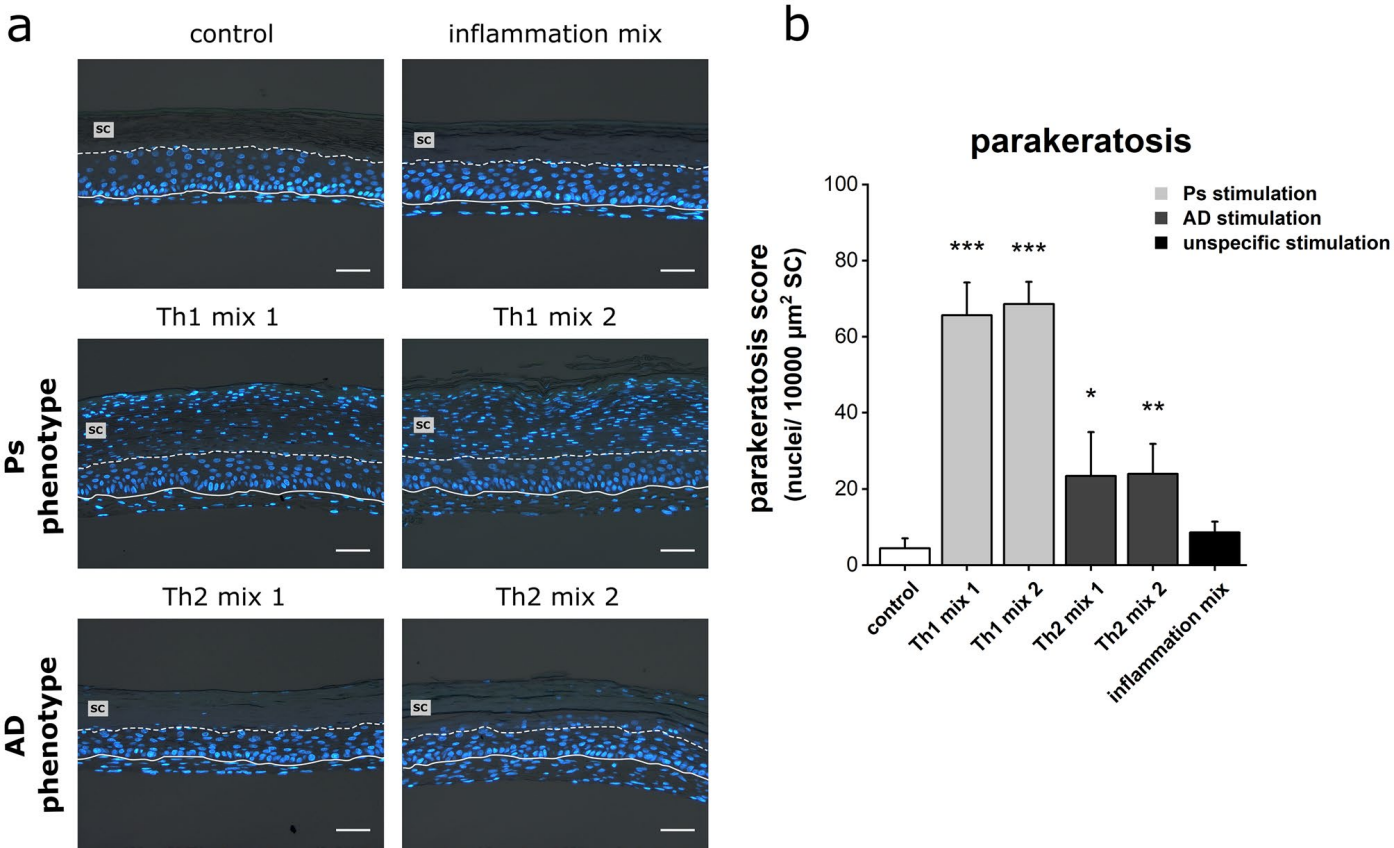
Pathological signs of parakeratosis in disease-associated skin models. (**a**) Nuclei of sections obtained from skin equivalents (unstimulated physiological control or stimulated with Th1 or Th2 cytokine mixes) were visualized using DAPI fluorescence staining. The solid line marks the basal membrane as dermal-epidermal interface and the dashed line indicates the *stratum corneum* (SC) layer above. Scale bar: 50 µm, (**b**) Quantification of nuclei per 10,000 µm^2^ SC area given as parakeratosis score (mean + SD) of n = 2 experiments with a total of 4 skin models (keratinocytes derived from two different donors). Statistics: one-way ANOVA with Dunett‘s T3 post hoc test; *p* ≤ 0.05 *, *p* ≤ 0.01 **, *p* ≤ 0.001 *** compared to control.

The localisation of nuclei and nuclear remnants within the cutaneous layers was monitored by DAPI fluorescence staining. Unstimulated controls showed a physiological distribution of nuclei underneath the *stratum corneum* and barely exhibited nuclear signals in the horny layer. If nuclei were detected in the cornified layer, they were always located close to the interface of granular and horny layer (Fig. 5a). Similar results were obtained for skin models stimulated with the inflammation mix indicating a physiological phenotype for nuclear localisation despite cytokine stimulation (Fig. 5a). Using specified cytokine cocktails (Th1 mix 1 and 2) for Ps induction, a pathological shift of the nuclear distribution towards upper layers of the *stratum corneum* was found. DAPI staining revealed a high number of nuclei residing in the horny layer of these diseased models. Th2-mediated AD induction also caused nuclear retardation within the cornified area. Compared to Ps models, the parakeratotic sign was, however, less pronounced in AD models (Fig. 5a). Quantification of the nuclei present in the *stratum corneum* emphasized the severe parakeratosis found in Ps models. The parakeratotic score obtained for AD models was also significantly higher compared to the unstimulated healthy control (Fig. 5b).

### Increased apoptosis in AD-like skin models

**Figure 6:**
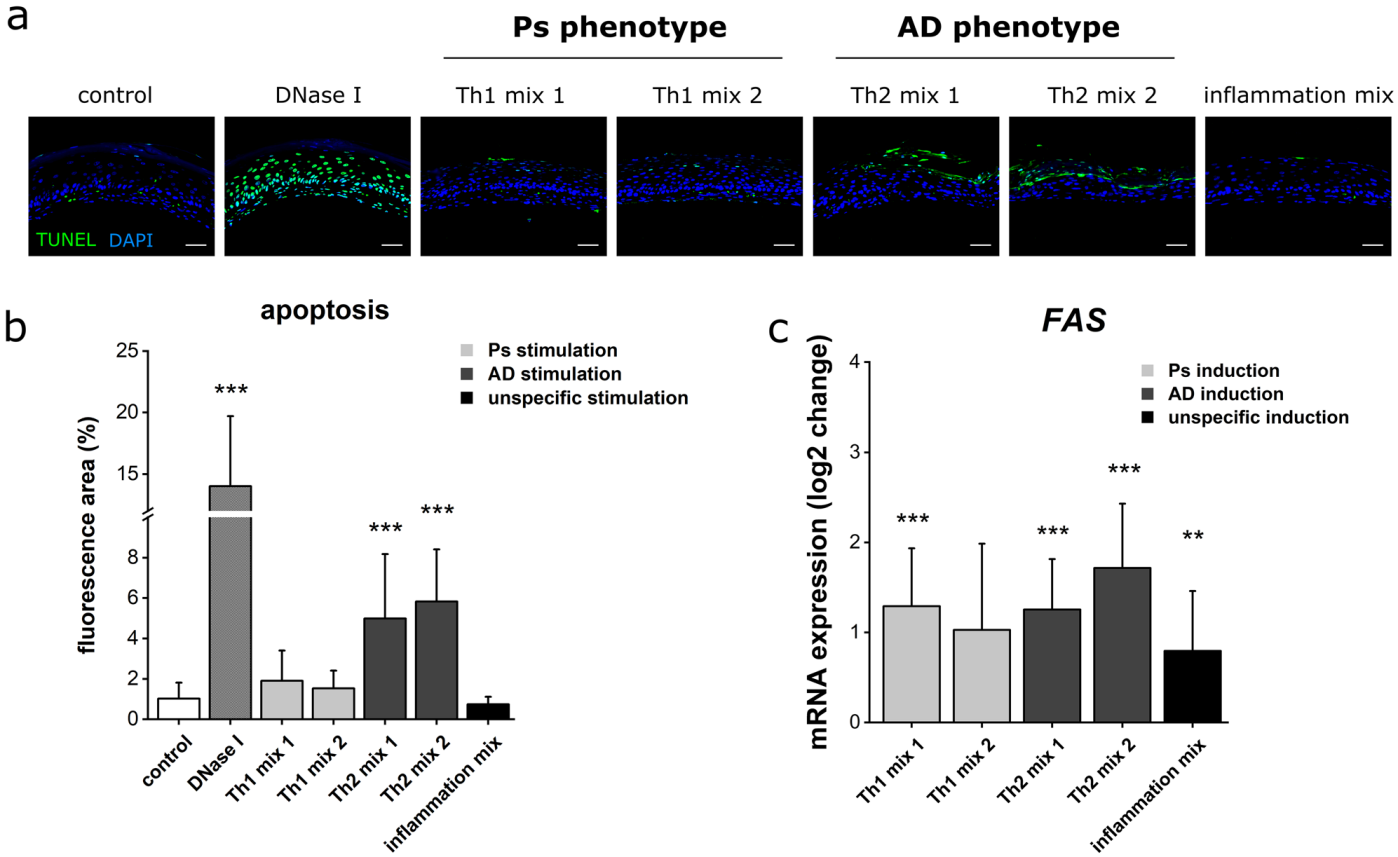
Increased apoptotic signs in AD-like skin models. (**a**) Apoptotic areas within skin equivalents (unstimulated physiological control or stimulated with Th1, Th2 cytokine mixes) were visualized using TUNEL staining (green fluorescence). DAPI was used for nuclear staining (blue fluorescence). (**b**) Quantification of apoptotic TUNEL fluorescence area (mean + SD) of n = 2 experiments with a total of 4 skin models (keratinocytes derived from one donor) presented as % per skin model. Statistics: Mann–Whitney U test; *p* ≤ 0.05 *, *p* ≤ 0.01 **, *p* ≤ 0.001 *** compared to control. (**c**) Log2 change of mRNA expression of the *FAS* cell death receptor gene was determined by real-time qPCR. Values are mean + SD of n = 2 experiments with a total of 4 skin models. Statistics: Mann–Whitney U test; *p* ≤ 0.05 *, *p* ≤ 0.01 **, *p* ≤ 0.001 *** compared to control.

Apoptotic features within skin layers of the models were screened using a TUNEL assay. Here, cell death was visualized by incorporating fluorescein-12-dUTP to terminal free 3’–OH ends of fragmented DNA. A method validation was provided by DNase I treatment causing DNA cleavage, thereby resulting in detection of a high number of apoptotic cells. Physiological control models rarely showed apoptotic signals. Stimulation with Th1 mixes or inflammation mix did not increase the number of cell death events. However, induction of AD-like phenotypes by Th2 stimulation triggered the occurrence of stained regions of fragmented DNA in upper layers of the epidermis (Fig. 6a). Due to this areal phenomenon, a single-cell counting of apoptotic cells was not applicable and therefore replaced by quantification of the cell death fluorescence area within the recorded area of each skin model. By doing so, a significant increase of the apoptotic signal was obtained for AD-like skin equivalents. Stimulation with Th1 and inflammation mixes was confirmed not to significantly affect apoptosis compared to healthy controls (Fig. 6b). For further insights into apoptosis, the mRNA expression of the *FAS* gene encoding a cell surface death receptor was analysed. *FAS* transcription was not only increased in AD skin models but also under Th1 stimulation for Ps induction and inflammatory TNF-α and INF-γ supplementation (Fig. 6c).

## Discussion

The development of three-dimensional skin models represents a reliable tool for modern in vitro research to reduce redundant animal testing. Cutaneous diseases such as psoriasis and atopic dermatitis can be induced in these 3D systems of human tissues in various ways. The usage of a representative model system is therefore a crucial decision based on the knowledge of the respective skin substitute and their limitations.

This study introduces and intensely characterizes new in vitro models for Ps and AD consisting of a dermal compartment based on a fibroblast-derived matrix and epidermal layers of primary keratinocytes. Since both, Ps and AD, affect the epidermal layer, several studies rely on the usage of RHE models solely focussing on the epidermal compartment^22–25,27,39^. However, crosstalk between keratinocytes and fibroblasts plays a major role in skin physiology and even modulates differentiation processes and molecular signalling pathways within the epidermis^40^. In addition, recent findings underline the participation of dermal fibroblasts in skin diseases including Ps and AD^28–31^. In the models developed in this study, the fibroblasts produce their own extracellular matrix. ECM components such as collagens, fibrin, proteoglycans and matricellular proteins are essential for skin homeostasis, provide a mechanical scaffold but are also involved in pathological processes of the skin such as infection and inflammation^41–43^. Our skin models showed a well-layered reconstruction of the skin with a basic dermal compartment and a multi-layered differentiated epidermis terminating in a horny layer. Primary keratinocytes possess the ability to differentiate upon calcium supplementation^44^. Hence, they are more suitable for the generation of 3D skin models than immortalized cell lines such as HaCaT keratinocytes, which are rather unresponsive to differentiation induction and consequently inferior in 3D tissue engineering^45–48^. The histological findings demonstrate differentiation defects by decreased levels of CK10 and loss of FLG in both, the Ps and AD substitutes. The mRNA transcripts of the differentiation marker genes *FLG*, *LOR* and *IVL* were significantly downregulated upon Th1 and Th2 stimulation. These findings indicate that the T-cell-associated cytokines used in the stimulation cocktails directly act as transcriptional modulators, thus, leading to decreased formation of differentiation proteins. Impaired differentiation is a key attribute of both skin disorders. Reduced mRNA and protein levels of the differentiation markers filaggrin and loricrin were seen in lesional skin of Ps and AD patients^49,50^. The Ps-associated cytokines IL-17A and IL-22 have already been shown to directly downregulate the *FLG*, *LOR* and *CK10* gene expression^51,52^. Suppression of *FLG* and *LOR* gene expression was also observed upon AD-related IL-13 and IL-4 stimulation in other studies^10,53^. Our in vitro models confirm these findings. A reduction of IVL protein levels was reported for AD skin and in vitro experiments evidenced diminished *IVL* mRNA levels of human keratinocytes in presence of IL-4 and/or IL-13^50,53^. Interestingly, despite potently declined *IVL* mRNA levels, the protein expression was barely affected in our Ps skin equivalents. IL-17 and IL-22 are known to downregulate *IVL* gene expression in vitro^54–56^. Since IL-17A and IL-22 were contained in the Th1 mixes, this might explain the reduced *IVL* transcription levels of the Ps models. Despite IVL being a late differentiation marker, the protein is usually reported to be increased in in vivo and in vitro psoriatic skin^27,57,58^. The findings of this study completely correlate with the results of an mRNA downregulation but protein induction observed by Rabeony et al.^27^. The role of involucrin in psoriasis is still not completely understood and provides controversial results. Our results fit regarding the cytokine effects on transcriptional level whereas the absent reduction in protein levels correlates with the in vivo reports^57,58^. The *IVL* mRNA downregulation was not transferred to protein expression, which might suggest a post-transcriptional modulation. IVL seems to be different from other differentiation markers in psoriatic lesions. Previous research has shown an altered distribution of IVL in the epidermal layer as a sign of disturbed differentiation and cornification in psoriasis^58,59^. In our study, IVL was detected in all suprabasal layers without localization differences between control and Ps equivalents indicating a limitation of this in vitro model compared to in vivo conditions. Unlike CK10, CK16 was induced in both, Ps and AD models. CK16 overexpression functions as a barrier alarmin associated with abnormal differentiation, hyperproliferation, tissue damage and inflammation^22,60,61^. Hence, it is no surprise that the inflammatory keratin is upregulated in skin diseases such as Ps and AD^12,19,62^. Other in vitro models of inflammatory skin disorders also showed a cytokine-driven increase in CK16 levels, thereby supporting the present findings^63,64^. An alteration of the Th1 and Th2 cytokine environment directly correlates with a shift in keratin protein expression with a reduction of physiological CK10 towards an induction of damage-associated CK16. Interestingly, another in vitro model of AD failed to detect a CK16 induction^10^. This underlines the advantage of using a thin fibroblast-derived matrix over of a thick rat collagen-based dermis, which is probably less permeable and lacks human ECM components.

A major difference between our Ps and AD models was observed in AMP production. S100A7 protein levels were markedly increased in Ps models while downregulated in AD substitutes. Similar findings were obtained on mRNA expression levels. Apart from the *S100A7* gene, other AMP genes such as *DEFB4*, *PI3* and *LCN2* showed comparable expression patterns. These findings correspond to typical hallmarks of both diseases. Previous studies have shown an AMP upregulation in patients with psoriasis^12,65^. Especially *DEFB4*, *S100A7* and *PI3* are among highly upregulated genes found in psoriatic plaque biopsies^66^. Once again, altered gene expression results from excessive in situ Th1/Th17 cytokine levels. The presence of IL-17A, IL-22 or TNF-α leads to an induction of *S100A7* mRNA expression^51,67^. *DEFB4*, *PI3* and *LCN2* genes are upregulated in response to IL-17A^16^. While an AMP upregulation is also observed in the chronic phase of AD, which is driven by the participation of Th1 and Th17 cells^13,68^, the present study focusses on the induction of the acute phase of AD via stimulation with solely Th2-derived cytokines. In this phase, AMPs are potently downregulated^13^. IL-4 and IL-13 have been shown to interfere with mRNA expression of beta-defensins by direct downregulation via STAT6 signalling^69^. Here, Th2 cytokines also inhibit AMP mRNA expression of *DEFB4*, *S100A7*, *PI3* and *LCN2* while Th1-derived cytokines act as transcriptional enhancer for AMP genes.

AD and Ps include a severe dysfunction of the epidermal barrier due to abnormal differentiation, inflammation and loss of structural proteins^70,71^. Penetration of the Lucifer Yellow dye revealed distinct barrier defects in the Ps and AD models developed in this study corresponding to clinical disease features. The horny layer was more permeable compared to the physiological control. Interestingly, this was not observed using an unspecific inflammatory cytokine mix containing TNF-α and INF-γ. This finding indicates that typical Th1/Th17-associated cytokines such as IL-17A, IL-6, IL-22 and IL-1α or Th2-derived cytokines like IL-4, IL-13 and IL-31 are causative for the disturbed barrier formation and not inflammation alone. TNF-α and INF-γ can be classified as Ps-related cytokines important for inflammation progression^72^. Even though, they do not seem to be the main drivers of the disease onset and barrier disruption in absence of IL-17A, IL-22 and IL-6. The observed barrier defects certainly result from the loss of FLG and other structural proteins. FLG plays a pivotal role in skin barrier homeostasis^73,74^. However, a study using FLG-deficient keratinocytes discovered no detrimental effects on barrier function and permeability^75^. This suggests that sole filaggrin depletion is not suitable to study barrier impairments as part of inflammatory diseases in vitro. Due to the multi-orchestrated conditions of inflammatory skin diseases, the barrier impairments seem to be a result of various factors. Hence, it is more advantageous to induce the disorders by cytokine stimulation to mimic T-cell-derived imbalances as the main course of disease development. Another aspect that comes into play is the effect on cell–cell contacts. IHC-staining of the desmosomal DSG1 transmembrane protein and the tight junction component CLDN1 demonstrated a distinct reduction of these cell adhesion molecules upon Th1 and Th2 stimulation. This might contribute to the observed barrier defect of the disease-associated skin equivalents. The cell–cell-contact genes *DSG1* and *TJP1* were downregulated in Ps and AD models as well, thereby confirming that the loss of cellular adhesion components already takes place on transcriptional levels. In contrast to the observed CLDN1 protein loss, the mRNA levels of the *CLDN1* gene appeared to be marginally affected upon Th2 stimulation. This indicates a posttranscriptional modulation leading to decreased CLDN1 protein production. These findings are in accordance with reduced cellular adhesion seen in Ps and AD skin in vivo^76–79^. Attenuation of cellular attachment proteins has been described for lesional skin of AD patients including a distinct loss of DSG1, CLDN1 and ZO-1 (TJP1) proteins^76,77,79^. An aberrant CLDN1 protein production was also observed in psoriatic plaques^78,80^. Increasing levels of IL-17 have been shown to correlate with diminishing CLDN1 and CLDN4 protein levels. These results state the potent influence of the cutaneous cytokine environment on regulatory affairs of tissue homeostasis and integrity. Interestingly, alterations of desmosomal proteins have been poorly investigated in terms of Ps. A study of Moreno-Sosa et al*.* revealed a downregulation of several genes encoding desmosome proteins such as *DGS4*, *DSG2*, *PKP2* (plakophilin-2), *DSP* (desmoplakin) and in a less potent manner also *DSG1*. Together with these findings, our study suggests an involvement of desmosomal proteins downstream to psoriatic Th1/Th17 cytokine signalling.

Inflammation is a major hallmark of both, Ps and AD^81^. Hence, the secretion and gene expression of pro-inflammatory mediators was measured in this study. The Ps and AD substitutes secreted significantly higher cytokine levels, especially of IL-8 verifying the inflammatory phenotype of the diseased skin models. In addition, *CXCL8* mRNA levels were increased under Th1 and Th2 environments. IL-8 plays a crucial role in inflammatory skin disorders, especially in AD where it correlates with disease severity^82^. High levels of IL-6 secretion were measured in the Ps skin equivalents resembling the elevated levels of the interleukin found in psoriatic skin^83^. A striking difference between the Ps and AD models was observed regarding the *IL1B* gene expression with increased levels upon Th1 but diminished transcripts upon Th2 stimulation. IL-1β is deep-rooted in the pathogenesis of Ps and upregulated in the lesional skin^84^. In contrast, a downregulation due to Th2 incubation is certainly a result from the ability of IL-4 to inhibit *IL1B* transcription^85^.

Hyperproliferation of epidermal keratinocytes in response to inflammatory processes is a fundamental clinical characteristic of Ps^86^. An increased number of basal keratinocytes with a positive signature for the mitotic marker Ki67 was identified in our Ps models due to Th1/Th17 stimulation. Hence, it can be concluded that our established Ps model is able to mimic the hyperproliferative state of Ps and that augmented mitotic events in the basal layer are a consequence of excessive Th1/Th17 cytokine levels. Especially IL-22 has been described to be a main inducer of keratinocyte proliferation^26,87^. IL-6, which was contained in the stimulation mixes as well as secreted in high levels by the skin models themselves, can act as a mitogenic signalling molecule, which further facilitates epidermal hyperproliferation in Ps^83^. The hyperproliferative character of our Ps equivalents can be considered a useful advantage since other in vitro models sometimes lack this hallmark feature^32,63,64^. There is a close proximity of the epidermal layers of our models to the cytokines in the medium below due to the thin self-established dermal matrix in between. This aspect probably accounts for a better cytokine availability and high activity on the keratinocytes leading to the development of typical disease-related attributes such as hyperproliferation.

Another pathological sign of both, Ps and AD skin, is the retention of nuclei in the *stratum corneum* known as parakeratosis^88,89^. The Ps equivalents showed a severe grade of parakeratosis in the horny layer while a moderate form was observed in the AD substitutes. Therefore, another disease-related feature was reproduced in our models. Other in vitro models were able to show the parakeratosis sign as well supporting the assumption that the retention of nuclei is a reliable attribute to detect pathological processes caused by abnormal epidermal differentiation^32,90^. In this aspect, these in vitro models are even superior to mouse models with xenotransplantation of psoriatic skin lacking the parakeratotic feature^91^.

On transcriptional levels, typical genes associated with AD were confirmed by upregulation of *NELL2*, *CA2* and *CCL26* in the Th2-stimulated skin equivalents. Even though the roles of CAII and NELL2 in AD are still not clear, they have been identified as reliable upregulated biomarker genes of AD allowing a clear molecular differentiation from Ps^19,92^. In vitro experiments have shown that *NELL2* and *CA2* are highly upregulated in response to Th2 cytokines like IL-4 and IL-13^24,93^. The CAII enzyme is responsible for pH regulation of the skin and involved in water transport and ion homeostatsis^92^. Since the enzyme catalyses a reaction leading to acidification, there seems to be a link to the increased pH value found in the skin of AD patients^94^. Eotaxin-3/CCL26 is another biomarker for AD. Elevated levels have been measured in AD patients, correlating with disease severity^20^. The chemokine is mainly upregulated by IL-4 as shown by the in vitro studies of Bao et al.^95^.

Apoptosis is one aspect that rarely gets attention during characterization of Ps and AD in vitro models. Our AD models showed a higher signal for DNA fragmentation, a main feature of cell death. This finding was similar to the increased number of TUNEL-positive cells found in an RHE model upon Th2 stimulation^96^. Concomitant increase of the *FAS* cell death receptor gene expression supported the apoptotic phenotype of the AD models. Fas-mediated apoptosis of epidermal keratinocytes is a critical issue seen in acute AD skin^97^. INF-γ has been shown to modulate Fas-mediated apoptosis signalling^98^. In contrast, our skin models stimulated with a combination of TNF-α and INF-γ did not show an elevated apoptosis rate via DNA cleavage but induction of *FAS* gene expression. Hence, it should be taken into consideration whether Fas-mediated apoptosis solely relies on INF-γ-induced *FAS* upregulation. On the other hand, Collins et al*.* described that DNA fragmentation occurs in terminal apoptosis and is not always linked to each other^99^. Likewise, the Ps skin equivalents did not show an alteration of apoptotic DNA fragmentation as revealed by TUNEL staining. However, there was an increase in *FAS* mRNA transcripts. The *FAS* gene has been found upregulated in patients suffering from Ps^100^. This finding was initially confusing since psoriatic keratinocytes are known to be rather apoptosis-resistant^101^. Gilhar et al*.* discovered an alternative pathway of Fas signalling leading to production of pro-inflammatory mediators such as IL-8 and TNF-α^102^. This hypothesis was supported by the finding of increased expression of anti-apoptotic factors like Bcl-2 and Bcl-x synchronous to elevated Fas levels in Ps^103,104^. This is indicative of a counter-regulatory mechanism of Fas-mediated apoptosis that prevents the psoriatic keratinocytes from cell death and converts the Fas signalling towards inflammation progression instead.

**Figure 7:**
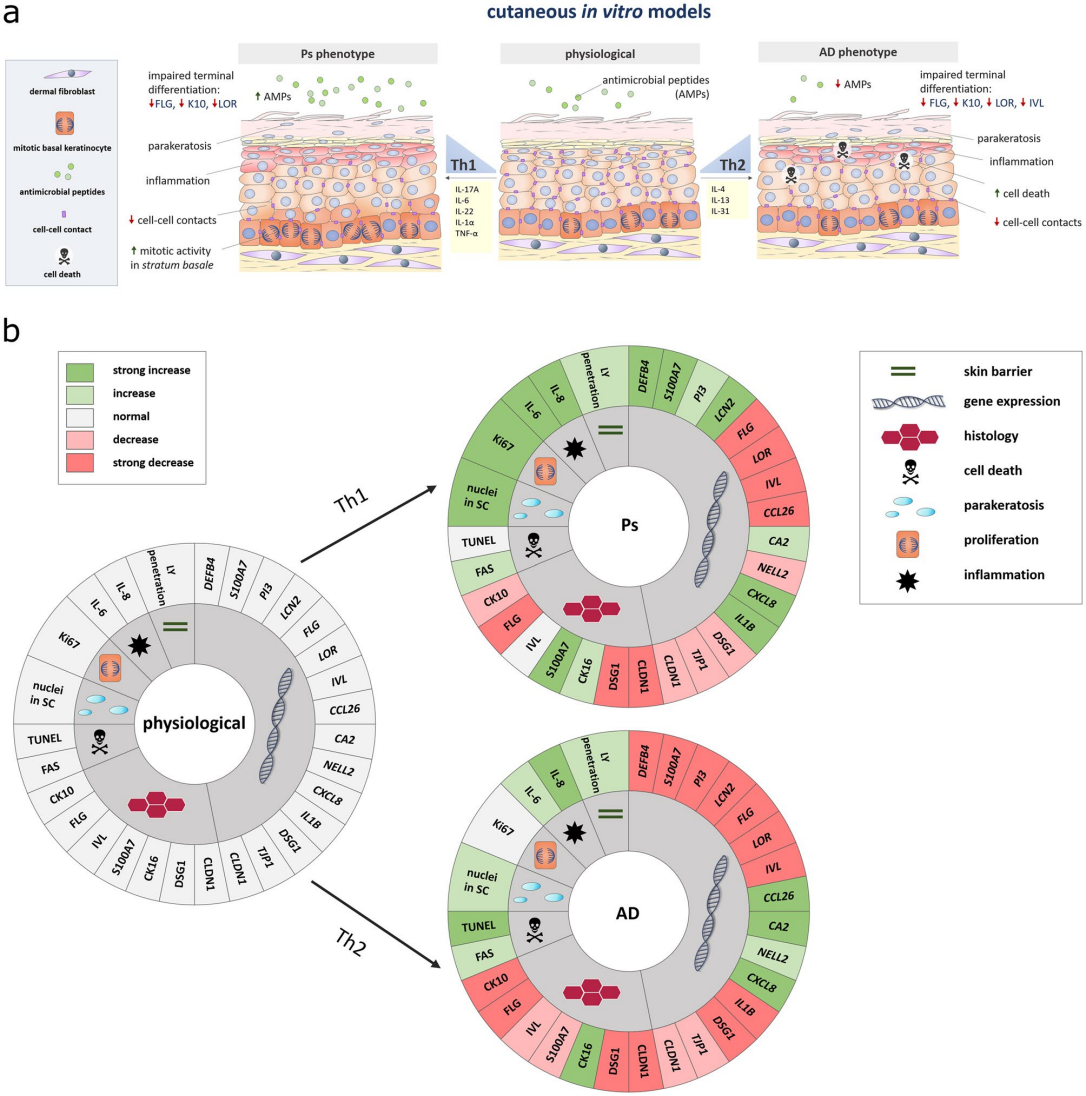
Characterization of Ps- and AD-like skin models (**a**) Graphical summary showing disease-associated attributes of the cutaneous in vitro models mimicing Ps or AD phenotypes. Ps skin models are characterized by differentiation defects, inflammation, impaired cellular attachment, hyperproliferation, parakeratosis and increased AMP production. AD skin models feature differentiation defects, inflammation, diminished cellular attachment, apoptotic events, parakeratosis and decreased AMP formation. (**b**) Color code-based semiquantitative assessment of investigated parameters. Abbrevaitions: SC—*stratum corneum*, LY—Lucifer Yellow, TUNEL—dT-mediated dUTP nick-end labeling.

In conclusion, the models established in this study represent a reliable tool to study AD and Ps in vitro. The fibroblast-derived matrix allows optimal diffusion of Th1 and Th2 cytokines to act on the epidermal layer as proximal regulators. This kind of model enables the crosstalk of fibroblasts and keratinocytes and provides an ECM of human proteins without addition of animal-derived collagen. Furthermore, there is no limitation of the cellular material as it has to be taken into account when using models based on diseased donor cells or patient biopsies for DED generation. The models display various features related to Ps and AD such as abnormal differentiation, loss of structural proteins, inflammation, hyperproliferation (Ps), parakeratosis, disease-associated gene expression patterns and apoptosis (AD) (Fig. 7a, b). The in-depth characterization of various molecular aspects allows a target-oriented use of these in vitro skin equivalents. Future studies will prove the potential of these models for a deeper understanding of the signalling processes in these inflammatory diseases or as in vitro tools for the testing of new therapeutic options.

## Materials & methods

### Generation of 3D skin models and induction of disease phenotypes

Cells and skin models were cultured at 37 °C with 5% CO_2_ under a humidified atmosphere. Dermal fibroblasts (PELO Biotech) were maintained in 175 cm^2^ cell culture flasks (Greiner Bio-One) in Dulbecco's modified Eagle's medium (DMEM; AMIMED® BioConcept Ltd.) supplemeted with 2% fetal calf serum (FCS; PAN Biotech), 5 μg/mL recombinant human insulin (AMIMED® BioConcept Ltd.), 5 ng/mL recombinant human fibroblast growth factor (Cellsystems) and 50 µg/mL gentamicin (Thermo Fisher). Primary keratinocytes isolated from juvenile foreskin were cultured using the keratinocyte growth medium 2 kit (KBM; PromoCell) with 50 µg/mL gentamicin. Primary keratinocytes were obtained and handled in accordance with relevant guidelines and regulations provided and approved by the Ethics Committee of the Medical Faculty of the Friedrich Schiller University Jena (4739-03/16). Informed consent was obtained from the legal guardians of all the donors of primary keratinocytes in the study.

Skin models were generated in inserts with 0.4 µm pore size sitting in a 12-well plate (Greiner Bio-One). For dermis generation, fibroblasts were trypsinized (trypsin–EDTA; Thermo Fisher) and 1.5 ∙ 10^5^ cells were seeded per insert in a submerse medium consisting of DMEM with 10% FCS, 50 µg/mL gentamicin and 150 µg/mL ascorbic acid (Sigma Aldrich). Dermal generation required 21 days of cultivation with regular medium exchange every 2–3 days inside the insert and the well underneath. Then, the addition of an epidermal layer was prepared by removing the medium and covering the dermal surface with 50 µg/mL fibronectin (PromoCell). Early passages of primary keratinocytes were thawed, resuspended in KBM submerse medium with 5% FCS, 50 µg/mL gentamicin and 150 µg/mL ascorbic acid and an amount of 13.5 ∙ 10^4^ cells was added per insert. After an incubation of 45 min in the CO_2_ incubator for cellular attachment, KBM submerse medium was added on top of the developing skin model and in the well underneath. Medium was exchanged 2 days later and Ca^2+^ concentration was increased by addition of 1.88 mM CaCl_2_ (Serumwerk Bernburg AG) to the medium after 5 days of epidermis development. At day 7 of epidermal formation, the medium on top was removed and the inserts were transferred to 12-well ThinCert® plates (Greiner Bio-One) for airlift cultivation (AL) of the skin models relying on medium supply from the bottom only. The medium used for AL incubation contained 1:1 DMEM + DMEM/F-12 (Thermo Fisher) with 5% FCS, 50 µg/mL gentamicin, 0.33 µg/mL hydrocortisone (Sigma Aldrich), 5 µg/mL transferrin (BBI Solutions), 5 µg/mL insulin (PELO Biotech), 3.99 ∙ 10^6^ ng/mL tri-iodothyronine (Sigma Aldrich), 13.51 µg/mL adenine (Sigma Aldrich), 1.88 mM CaCl_2_ and 150 µg/mL ascorbic acid. During AL cultivation, skin models were primed for Ps and AD via addition of recombinant cytokine cocktails at day 0, 2, 5, 7 and 9 during medium exchange. For Ps induction Th1 mix 1 with 10 ng/mL IL-17A, 10 ng/ml IL-6, 25 ng/mL IL-22, 10 ng/mL IL-1α (7Bioscience GmbH) and Th1 mix 2 consisting of Th1 mix 1 plus 10 ng/mL TNF-α was used. AD was induced with Th2 mix 1 (50 ng/mL IL-4, 50 ng/mL IL-13) or Th2 mix 2 (Th2 mix 1 plus 25 ng/mL IL-31). Stimulation with 10 ng/mL TNF-α and 5 ng/mL INF-γ served as unspecific inflammation control. After 12 days of AL, skin models completed differentiation and cornification.

### Haematoxylin & eosin staining

Histological preparation of the skin equivalents and HE staining was performed as described previously^105,106^. Briefly, skin equivalents were placed in embedding cassettes (Kabe Labortechnik) and fixed in 4% formalin (Dr. K. Hollborn & Söhne). Sections of paraffin-embedded samples were placed onto glass slides (Menzel) and deparaffined. Deparaffination (using xylene), rehydration (via decreasing alcohol series), HE staining and final dehydration (via increasing alcohol series; xylene) before mounting was performed automatically with the Leica Autostainer XY (Leica).

### Immunohistological staining

Skin equivalents were placed in embedding cassettes (Kabe Labortechnik) and fixed in 4% formalin (Dr. K. Hollborn & Söhne). Sections of paraffin-embedded samples were placed onto glass slides (Menzel) and deparaffined. For specific protein detection, the following primary antibodies were used: anti-CK10 (Ventana Medical Systems), anti-FLG, anti-IVL, anti-S100A7, anti-CK16, anti-DSG1 (Thermo Fisher), anti-Ki67 (Agilent Technologies). The ultraView Universal Alkaline Phosphatase Red Detection Kit (Ventana Medical Systems) was used for detection according to manufacturer’s instruction. An automated staining procedure was performed using the BenchMark GX (Ventana Medical Systems). IHC staining of S100A7, cytokeratin-16 and desmoglein-1 was executed manually according to manufacturers’ recommendations.

For mitosis quantification, Ki67-positive cells within the *stratum basale* were counted via ImageJ and calculated in % relative to unstimulated physiological control which was set as 100% regarding mitotic events. Data were derived from n = 2 experiments with a total of 4 skin models and two pictures per sample were taken and quantified.

### ELISA-based detection of cytokine secretion

Release of the pro-inflammatory mediators IL-6 and IL-8 in the undernatants of the skin equivalents was measured using the Human IL-6 ELISA development kit (Mabtech) and IL-8/CXCL8 DuoSet ELISA (R&D Systems). Kit instructions provided by the manufacturers were followed accordingly. Absorbance measurement at 450 nm and reference reading at 620 nm was performed with the SPECTROstar Omega microplate reader (BMG Labtech). Calculation of the cytokine concentrations was done by a four-parameter fitting with linear scaling for OD values and logarithmic scaling for the concentrations. A Th1 correction was conducted for the IL-6 analysis of Th1-treated skin models to ensure the presentation of endogenous IL-6 release from the skin models. The sole Th1 mixes containing recombinant IL-6 were incubated in an additional well plate and IL-6 levels measured via ELISA were subtracted from the corresponding values obtained for the Th1-treated skin models. Data were derived from n = 2 experiments with a total of 8 skin models. Technical duplicates per sample were measured.

### Gene expression analysis

RNA from the skin equivalents was extracted using the RNeasy Mini kit (Qiagen). Skin models were lysed in RLT buffer containing 10 µL/mL 2-mercaptoethanol and mechanically dissected by 3 mm steal beads at 30 Hz in a mixer mill (Retsch). Proteinase K (200 µL/mL; AppliChem GmbH) was added and samples were incubated at 55 °C for 10 min at 1000 rpm. RNA isolation from tissue lysates was performed automatically via the QIAcube robotic workstation. Genomic DNA was digested using the DNase I, RNase-free kit (Thermo Fisher) according to manufacturer’s instructions. RNA concentration was measured and cDNA was synthesized by reverse transcription based on the High Capacity cDNA Reverse Transcription Kit by Applied Biosystems (Thermo Fisher) following the manual recommendations. For quantitative real-time PCR (qPCR), the QuantiNova™ SYBR Green PCR Kit (Qiagen) was used. Samples were diluted to 0.5 ng/µL. The qPCR reaction mix contained 1 × of the SYBR Green master mix, 500 nM forward and reverse primer each, 3 µL of the diluted cDNA and up to 20 µL PCR grade water. PCR reaction was performed in the qTOWER^3^G (Analytik Jena) with the following protocol: 95 °C for 3 min (initial thermal activation of polymerase), 40 cycles of 95 °C for 5 s (denaturation), 57 °C for 10 s (annealing), 72 °C for 10 s (elongation). Relative expression was calculated according to the primer efficiency-corrected algorithm described by Pfaffl et al*.*^107^ using *ACTB* gene expression as housekeeping reference. Non-detects were handled in accordance with Goni et al*.* where undetermined values were set to the Ct maximum 40 while absent values were imputed to the median of detected replicate values^108^. Expression levels are presented as log2 change with a log_2_ (2) = 1 implying a twofold upregulation and log_2_ (0.5) = − 1 indicating a downregulation by a factor of 2. Data were derived from n = 2 experiments with a total of 4 skin models. Technical duplicates per sample were measured. Primer sequences are shown in Table 1.

**Table 1:** Primer sequences and purchased primer sets.

Gene name	Primer sequences (5′ → 3′)	
	Forward	Reverse
*DEFB4*	TGATGTCCTCCCCAGACTCA	CCACCAAAAACACCTGGAAGAG
*DSG1*	TCCCCACATTTCGGCACTAC	GCCCAGAGGATCGAGAATAGG
*IL1B*	GGACAAGCTGAGGAAGATGC	CCATATCCTGTCCCTGGAG
*LCN2*	ATGTCACCTCCGTCCTGTTTAG	TAATGTTGCCCAGCGTGAAC
*NELL2*	AGCCAAAACATCAGCCAAGC	TTCCCTTCATGGTGCAAGTC
*S100A7*	GTCCAAACACACACATCTCACT	CATCATCGTCAGCAGGCTT
	**Commercially validated primer sets (Qiagen)**	
*ACTB*	Hs_ACTB_1_SG QuantiTect® Primer Assay	
*CA2*	Hs_CA2_1_SG QuantiTect® Primer Assay	
*CCL26*	Hs_CCL26_1_SG QuantiTect® Primer Assay	
*CLDN1*	Hs_CLDN1_1_SG QuantiTect® Primer Assay	
*CXCL8*	Hs_CXCL8_1_SG QuantiTect® Primer Assay	
*FAS*	Hs_FAS_1_SG QuantiTect® Primer Assay	
*FLG*	Hs_FLG_1_SG QuantiTect® Primer Assay	
*IVL*	Hs_IVL_1_SG QuantiTect® Primer Assay	
*LOR*	Hs_LOR_1_SG QuantiTect® Primer Assay	
*PI3*	Hs_PI3_2_SG QuantiTect® Primer Assay	
*TJP1*	Hs_TJP1_1_SG QuantiTect® Primer Assay	

Oligonucleotide sequences used for expression analysis of the genes *DEFB4* (defensin, beta 4), *DSG1* (desmoglein-1), *IL1B*, (interleukin-1 beta), *LCN2* (lipocalin-2), *NELL2* (neural epidermal growth factor-like like 2), *S100A7* (psoriasin) are listed in 5’-3’ direction. Validated primer sets were purchased from Qiagen to examine the gene expression of *ACTB* (beta-actin), *CA2* (carbonic anhydrase), *CCL26* (C–C motif chemokine ligand 26/eoatxin-3), *CLDN1* (claudin-1), *CXCL8* (C-X-C motif ligand 8/interleukin-8), *FAS* (Fas cell death receptor), *FLG* (filaggrin), *IVL* (involucrin), *LOR* (loricrin), *PI3* (peptidase inhibitor 3/elafin) and *TJP1* (tight junction protein-1).

### Lucifer Yellow penetration analysis

A Lucifer yellow (LY; Sigma Aldrich) solution of 1 mM in ddH_2_O was applied topically to the skin models. After 2 h of incubation in the CO_2_ incubator, superficial LY was removed by washing two times with 1 × PBS. Pre-treatment with 0.25% SDS 24 h prior to LY staining served as positive control. Formalin-fixed paraffin sections were placed onto Superfrost Plus (Menzel) slides and deparaffined. For combined nuclear staining and mounting, the Fluoroshield™ with DAPI histology mounting medium (Sigma Aldrich) was used. Data were derived from n = 2 experiments with a total of 4 skin models. For quantification, two pictures per sample were taken and measured via ImageJ.

### Parakeratosis screening

Formalin-fixed paraffin sections were placed onto Superfrost Plus (Menzel) slides and deparaffined. For nuclear staining and mounting, a DAPI-containing mounting medium was applied. Nuclei and nuclear remnants within the *stratum corneum* were counted and calculated as parakeratosis score relative to 10,000 µm^2^ of the recorded area of each horny layer. Data were derived from n = 2 experiments with a total of 4 skin models. For quantification, two pictures per sample were taken and measured via ImageJ.

### Apoptosis screening by TUNEL-based detection

Formalin-fixed paraffin sections were placed onto Superfrost Plus (Menzel) slides and deparaffined. For cell death detection, a DNA nick end-labelling technique was executed using the DeadEnd™ Fluorometric TUNEL system (Promega). Instructions provided by manufacturer’s user protocol were precisely followed. DNase I treatment prior to sample equilibration and TdT-mediated fluorescein labelling served as positive control. Unstimulated/physiological skin models were used for this purpose and stained in separated jars. Nuclear counterstaining was provided by the usage of a DAPI-containing mounting medium. Apoptotic area was calculated as percentage from the whole skin model area. Data were derived from n = 2 experiments with a total of 4 skin models. For quantification, two pictures per sample were taken and measured via ImageJ.

### Fluorescence microscopy

Slides generated for LY barrier penetration analysis, parakeratosis screening and TUNEL-based apoptosis detection were viewed under an Axio Scope.A1 fluorescence microscope (Carl Zeiss) and pictures were recorded via the AxioCam MRc camera (Carl Zeiss). Fluorescence of DAPI, LY and fluorescein-12-dUTP-labelled DNA (TUNEL assay) was visualized with appropriate filter sets (λ_ex_ = 358 nm for DAPI; λ_ex_ = 488 nm for LY and fluorescein).

### Graph preparations & statistical processing

Graphs were prepared using the OriginPro 2019 (OriginLab) software while statistical analysis was performed with IBM SPSS Statistics 26. Data were tested regarding normal distribution by Kolmogorov–Smirnov test. Given normal distribution, data were statistically analysed via one-way ANOVA with Bonferroni post hoc test for data sets with homgeneous variances or using the Dunett’s T3 post hoc in case of inhomogeneity of variances. The nonparametric Mann–Whitney U test was applied when normal distribution was rejected. Significant deviations were stated by *p* ≤ 0.05 *, *p* ≤ 0.01 **, *p* ≤ 0.001 ***.

## Supplementary Information

Supplementary Information.

## Data Availability

Data of this study are available from the corresponding author upon request. Gene expression data sets are available in the supplementary material.
