# Supplementary material for: Induction of psoriasis- and atopic dermatitis-like phenotypes in 3D skin equivalents with a fibroblast-derived matrix
Source: Sci Rep. 2023 Jan 31;13:1807. doi: 10.1038/s41598-023-28822-7 (PMC9889787; doi:10.1038/s41598-023-28822-7)
Supplement: Supplementary file 1 — Supplementary Information. [file 41598_2023_28822_MOESM1_ESM.docx]

**SUPPLEMENTARY**

**

**

**Supplementary Figure S1: Disease-associated gene expression patterns in Ps- and AD-like skin models.**

Log2 change of mRNA expression of genes encoding antimicrobial peptides (*DEFB4*, *S100A7*, *PI3*, *LCN2*), structural proteins (*FLG*, *IVL*, *LOR*), chemokines & cytokines (*CXCL8*, *IL1B*, *CCL26*), AD biomarkers (*CA2*, *NELL2*) and protein components of cellular adhesions (*DSG1*, *TJP1*, *CLDN1*) was determined by real-time qPCR. Values are mean of n=2 experiments with a total of 4 skin models.
